# Sexual Functioning and Depressive Symptoms in Levothyroxine-Treated Women with Postpartum Thyroiditis and Different Vitamin D Status

**DOI:** 10.3390/nu17132091

**Published:** 2025-06-24

**Authors:** Karolina Kowalcze, Joanna Kula-Gradzik, Anna Błaszczyk, Robert Krysiak

**Affiliations:** 1 Department of Pediatrics in Bytom, Faculty of Health Sciences in Katowice, Medical University of Silesia, Stefana Batorego 15, 41-902 Bytom, Poland; jkula-gradzik@sum.edu.pl; 2 Department of Pathophysiology, Faculty of Medicine, Academy of Silesia, Rolna 43, 40-555 Katowice, Poland; 3 Department of Pediatrics, Provincial Hospital in Częstochowa, PCK 7, 42-200 Częstochowa, Poland; 4 Department of Internal Medicine and Clinical Pharmacology, Medical University of Silesia, Medyków 18, 40-752 Katowice, Poland; rkrysiak@sum.edu.pl

**Keywords:** depressive symptoms, female sexual response, hormonal profile, hormone replacement therapy, hypothyroidism, thyroid autoimmunity

## Abstract

*Background/Objectives*: Hypothyroidism and thyroid autoimmunity have a negative effect on women’s sexual health, which is only partially reversed by thyroid hormone substitution. Sexual functioning in thyroid disorders after delivery has been poorly researched. The aim of our study was to compare the effect of levothyroxine on sexual response and depressive symptoms in women with postpartum thyroiditis (PPT) and different vitamin D status. *Methods*: The study population consisted of three matched groups of women with the hypothyroid phase of PPT: two groups with subclinical and one with overt thyroid hypofunction. Each group included similar numbers of women with normal and low vitamin D status. For the following six months, one group of women with subclinical hypothyroidism and all women with overt thyroid hypofunction received levothyroxine. At the beginning and at the end of the study, all participants completed questionnaires evaluating female sexual function (FSFI) and depressive symptoms (BMI-II). The remaining outcomes of interest included thyroid antibody titers, and the serum levels of 25-hydroxyvitamin D, TSH, free thyroid hormones, sex hormones, and prolactin. *Results*: Before levothyroxine substitution, women with overt and subclinical disease differed in the total FSFI score, all domain scores, and the overall BDI-II score. Within each study group, domain scores for desire were greater in women with vitamin D sufficiency than in those with vitamin D deficiency/insufficiency. Testosterone and estradiol levels were lower in women with overt than in women with subclinical hypothyroidism, while the opposite relationship was found for prolactin. Levothyroxine treatment improved all domains of female sexual function and reduced the total BDI-II score in both patients with overt and subclinical hypothyroidism and normal vitamin D status. In women with vitamin D deficiency/insufficiency, the impact of this agent was limited to arousal, lubrication, and sexual satisfaction. Levothyroxine replacement reduced thyroid antibody titers only in women with normal vitamin D status. The impact on testosterone was limited to women with normal vitamin D status, and was more pronounced in women with overt than subclinical disease. The effect on estradiol and prolactin, observed only in overt disease, was unrelated to vitamin D status. The increase in sexual functioning correlated with the following: 25-hydroxyvitamin D levels (in vitamin D-deficient/insufficient women); the impact on thyroid peroxidase antibodies, free triiodothyronine and testosterone (for desire and arousal); and the changes in the overall BDI-II score. Five years later, the quality of life was better in vitamin D-sufficient women receiving levothyroxine in the postpartum period. *Conclusions*: Low vitamin D status attenuates the impact of levothyroxine on female sexual function and depressive symptoms in women with the hypothyroid phase of PPT.

## 1. Introduction

It is well evidenced that hypothyroidism exerts a negative effect on women’s sexual functioning. The risk of female sexual dysfunction (FSD) was markedly increased in subjects with overt thyroid hypofunction and in individuals with subclinical thyroid hypofunction and thyroid-stimulating hormone (TSH) levels above 10 mIU/L [1]. In another study, this complication developed frequently (59%) in individuals with TSH concentration in the range between 4.5 and 10 mU/L [2]. Although both overt and subclinical disease were accompanied by lower values of the global Female Sexual Function Index (FSFI) score and all FSFI domain scores, these disturbances were more pronounced in overt disease [2,3]. Interestingly, women’s sexual health was worse if thyroid hypofunction was induced by Hashimoto thyroiditis than in individuals with hypothyroidism of non-autoimmune origin [2]. Because of the lack of dedicated studies, it is difficult to conclude whether the type of thyroid autoimmune disorder determines female sexual functioning.

Although adequate thyroid hormone substitution should theoretically restore female sexual response, the impact of levothyroxine on sexual functioning is not so clear-cut. In an Italian study, hypothyroid women receiving this hormone had lower values of the global score and all domain scores of the FSFI questionnaire than healthy controls. The global score was also lower in patients with this disorder than in women with diabetes and obesity [4]. In another Italian study, levothyroxine-treated women were characterized by impaired excitement and orgasm [5]. In a previous study of our research team, despite effective levothyroxine substitution, hypothyroidism induced by surgical removal of the thyroid gland was associated with a 50% risk of developing sexual dysfunction [6]. In a Spanish study, despite levothyroxine-induced normalization of TSH levels, sexual dysfunction persisted, involving all domains of the Questionnaire on Women’s Sexual Function: desire, arousal, lubrication, orgasm, penetration pain, sexual initiative, sexual communication, sexual activity, and general sexual satisfaction [7]. It is difficult to explain these findings. They do not seem to be associated with using too low doses of levothyroxine. In one study, average TSH levels were high normal (4.4 mIU/L) [4], while in the remaining ones they were in the range between 1.8 and 2.2 mU/L [5,6,7]. The relatively weak effects of thyroid hormone substitution cannot be also attributed to differences in the autoimmune status of patients, as they were observed in hypothyroidism of both autoimmune and non-autoimmune origin. Thirdly, they cannot be explained by the impact of concurrent disorders and concomitant therapies. A partial explanation for the limited effects may be imperfections of thyroid hormone substitution [6]. However, although replacing levothyroxine with levothyroxine/liothyronine combination therapy improved libido and arousal, the replacement had a neutral effect on lubrication, orgasm, sexual satisfaction, and pain [6]. Thus, it seems that there are additional factors that determine the strength of thyroid hormone action on women’s sexual health. There are no data on the association between the strength of levothyroxine action and the disorder causing thyroid hypofunction.

One of the reasons for the limited effectiveness of thyroid hormone substitution may be low vitamin D status, which in previous studies had an unfavorable effect on female sexual response. Sexual dysfunction was reported in as many as 72% of Turkish women with vitamin D deficiency and 48% of women with vitamin D insufficiency [8]. In reproductive-age Polish women, the risk of this complication was estimated at 36% in case of vitamin D deficiency and at 21% in case of vitamin D insufficiency [9]. Young Turkish patients meeting the diagnostic criteria of FSD were found to be characterized by lower 25-hydroxyvitamin D (25OHD) concentrations than women without sexual dysfunction [10]. Although impaired sexual functioning was reported even in women with mild disturbances in vitamin D homeostasis, the lowest domain scores were found in women with vitamin D deficiency [8,9]. The beneficial effect on sexual health in women with low vitamin D status in response to the administration of exogenous vitamin D was observed in different age populations. Vitamin D treatment of young women with euthyroid Hashimoto thyroiditis improved the global score and most domain scores of the FSFI questionnaire (except for orgasm and pain), and the effect on global sexual functioning, desire, and arousal was stronger than that of selenomethionine and myo-inositol [11]. In the same age group, exogenous vitamin D had a beneficial effect on all domains of the FSFI questionnaire [12]. Low vitamin D doses (300 IU daily) administered together with isoflavones, calcium, and inulin caused a significant reduction in vasomotor, physical and sexual domain scores of the Menopause-Specific Quality of Life questionnaire, and a significant increase in all FSFI domain scores in the fifth and sixth decade of life, and this effect was more pronounced than that of placebo [13]. Vitamin D suppositories administered intravaginally were superior to placebo in improving sexual functioning in sexually active postmenopausal women [14]. Lastly, the beneficial effect on excitement in this group of patients was induced also by topical vitamin D administration [15]. Unfortunately, to date, nothing is known about the association between vitamin D homeostasis and the sexual health of women with concurrent disorders.

Postpartum thyroiditis (PPT) is a destructive autoimmune disease developing within the first 12 months after delivery, or, in exceptional cases, after miscarriage or medical abortion in women who were euthyroid prior to pregnancy [16,17]. The estimated pooled prevalence in the general population is approximately 8%, varying markedly between different studies from 1.1 to 21.1% [18]. The disease seems to be caused by the return of Th1 mediated immunity, which is suppressed in pregnancy [19,20]. PPT may present as transient thyrotoxicosis, transient hyperthyroidism followed by usually transient hypothyroidism or isolated, transient or permanent, hypothyroidism [21]. Contrary to Hashimoto thyroiditis, lymphocytic infiltration of the thyroid gland in PPT is not accompanied by the presence of germinal centers, oxyphil cell metaplasia, and glandular fibrosis [22]. It seems that vitamin D status impacts the course of PPT. Women with this disorder were characterized by lower levels of 25OHD than their peers without thyroid pathology who gave birth within 12 months before the study [23], while the chronic administration of exogenous vitamin D to women with PPT reduced thyroid autoimmunity [24]. The results of both studies suggest the existence of a relationship between the degree of vitamin D deficit and the severity of PPT, similar to that observed in individuals with Hashimoto thyroiditis [25]. Moreover, vitamin D screening and supplementation policy may improve thyroid function in the postpartum period, as in the case of adverse pregnancy outcomes (pre-eclampsia, gestational diabetes mellitus, and preterm delivery) [26].

To better understand the association between vitamin D status and the impact of thyroid hormone replacement on women’s sexual health, the aim of our study was to investigate whether this vitamin determines the impact of levothyroxine treatment on sexual functioning in women with PPT. Moreover, because thyroid autoimmunity after delivery was suggested to be associated with impaired mood [27,28], the study compared the relationship between sexual functioning and depressive symptoms in response to levothyroxine substitution in women with different vitamin D status. Lastly, we assessed the consequences of levothyroxine treatment of PPT for quality of later life.

## 2. Materials and Methods

This study adhered to the ethical standards outlined in the 1964 Declaration of Helsinki and its subsequent amendments. All women provided written informed consent after receiving a detailed explanation of all procedures, and were informed that they could withdraw their consent at any time without negative consequences. The study protocol was approved by the appropriate ethics committee. The main study was performed in years 2016–2019, while the second part between 2021 and 2024 (5 years later).

### 2.1. Study Population

The study population was selected among white Polish Caucasian women (18–45 years old) with transient hypothyroidism secondary to PPT living in the Częstochowa district of the Silesian Voivodeship, the urbanized industrial region located in the southern part of Poland. The inclusion criteria which had to be met included the following: (a) titers of thyroid peroxidase antibodies (TPOAb) above 100 IU/mL; (b) the characteristic sonographic appearance of the thyroid gland (multifocal or diffuse thyroid hypoechogenicity but without atrophy and fibrosis); (c) serum TSH levels exceeding 4.5 mIU/L on two different occasions; and (d) the lack of clinical, laboratory, or sonographic abnormalities suggestive of thyroid disease before pregnancy. Women with autoimmune thyroid diseases diagnosed for the first time during gestation, post-partum Graves’ disease or the thyrotoxic phase of PPT; lactating women; and women who stopped breastfeeding within three months before the beginning of the study were not considered for enrollment. The remaining exclusion criteria were as follows: another chronic disease; psychiatric problems; bisexuality; sexual inactivity; gestation; inherited, congenital or acquired anomalies of the reproductive system; urogynecological operations that might affect sexual activity; and any chronic treatment (except for vitamin D supplementation). The patients were assigned to one of three groups. Two groups (groups I and II) included women with normal free thyroid hormone levels (free thyroxine between 10.8 and 21.3 pmol/L and free triiodothyronine between 2.2 and 6.5 pmol/L) (subclinical hypothyroidism), while the last group (group III) included women with free thyroxine below the lower limit of normal (overt hypothyroidism). Patients from groups I and III accepted levothyroxine replacement, while group II included women who were not interested in this treatment. Because of a small number of women with overt disease who were reluctant to be treated and owing to ethical issues, there was no group of levothyroxine-naïve women with overt thyroid hypofunction.

Depending on serum 25OHD levels, the participants were classified as vitamin D-sufficient (levels between 75 and 200 nmol/L or vitamin D deficient/insufficient (levels below 75 nmol/L) [29]. The former ones belonged to subgroup A, while the latter to subgroup B. For ethical reasons, individuals with 25OHD concentrations below 25 nmol/L were excluded, while women with a 25OHD concentration between 25 and 50 nmol/L were included only if they refused vitamin D supplementation.

**Figure 1:**
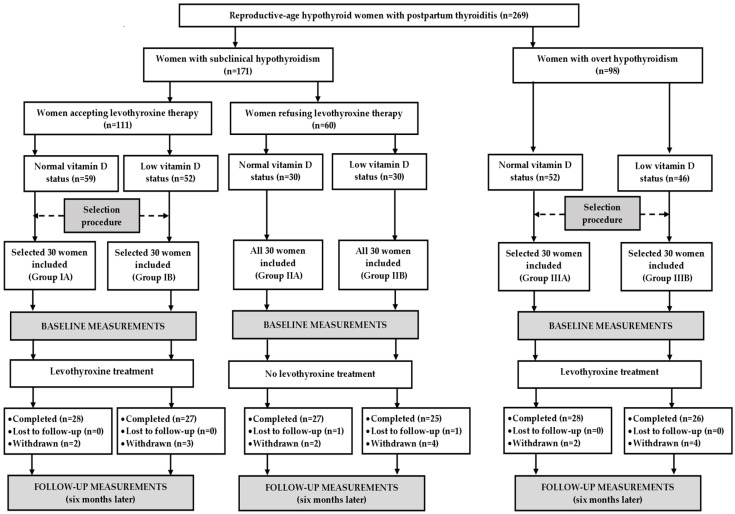
Flow the patients through the study.

An a priori sample size calculation showed that with an alpha of 0.05 and a power of 80% we would need 24 patients per group to demonstrate a 15% difference in the overall FSFI score, which was the study’s primary endpoint. Considering possible withdrawals and losses to follow-up, the study population was increased by 25% (to 30 patients per group). Because the outcome measures were found to be influenced by seasonal factors [30,31,32,33,34], similar numbers of patients were enrolled in each season: spring (n = 32 [27%]), summer (n = 29 [24%]), autumn (n = 28 [23%]) and winter (n = 31 [26%]). All women with subclinical hypothyroidism refusing levothyroxine treatment were included. The remaining groups were selected from larger populations of potential participants in order to create three study groups, which were matched for age, body mass index, and blood pressure (Figure 1).

### 2.2. Study Design

Throughout the study (six months), groups I and III received levothyroxine treatment, while group II remained levothyroxine-naïve. The starting dose of levothyroxine (50 μg daily) was in most patients increased in two-week intervals to the final dose, which was administered until the end of the study. The drug was taken as a single daily dose, on an empty stomach, one-half to one hour before breakfast (in order to avoid erratic absorption of levothyroxine). The final daily dose varied between 100 and 200 μg in patients with overt, and between 50 and 100 μg in patients with subclinical, hypothyroidism. The dose was adjusted to obtain serum TSH levels in the range between 0.4 and 2.5 mU/L. No changes in vitamin D supplements were allowed during the study. The dietary intake of vitamin D was calculated based on an analysis of dietary diaries completed by the participating women. The total intake was obtained by summing the dietary and supplemental intake. To limit the influence of pharmacokinetic interactions with levothyroxine, vitamin D supplements were taken at bedtime. Medication adherence was measured every two months by counting returned tablets. Adherence was considered satisfactory if the coverage of the prescribed medication was at least 90%. Other pharmacotherapies were allowed only if they were used for no more than ten days, but not during the last four weeks of the study. The withdrawal criteria were as follows: (a) other changes in the medication regimen, (b) consent withdrawal, (c) serious adverse effects, (d) sudden hospitalization and (e) poor medication adherence.

### 2.3. Laboratory Assays

All laboratory assays were performed before levothyroxine replacement and six months later. Venous blood samples were drawn from the antecubital vein in a quiet, temperature-controlled room, after overnight fasting. Blood samples were collected in the morning hours in the follicular phase of the menstrual cycle, after the patients had been resting for 30 min. To minimize the impact of pulsatility and venipuncture-induced stress [35,36], prolactin levels were assessed in three samples collected at 20 min intervals. All samples were immediately coded so that laboratory staff performing assays were blinded to the subjects’ clinical characteristics and study group assignment. All analyses were carried out in duplicate to ensure reproducibility and accuracy, and the results were averaged. Serum titers of thyroid antibodies (TPOAb and antibodies against thyroglobulin [TgAb]), as well as serum levels of 25OHD, TSH, free thyroid hormones, testosterone, estradiol, prolactin, and dehydroepiandrosterone sulfate (DHEAS) were assessed using acridinium ester technology (ADVIA Centaur XP Immunoassay System, Siemens Healthcare Diagnostics, Munich, Germany).

### 2.4. Questionnaires

Immediately after phlebotomy, all participants were asked to complete three questionnaires. During the questionnaire survey, neither the participants nor the researchers knew the results of laboratory tests. The investigators were not allowed to influence and interfere with the answers, and were trained not to offer personal opinions or advice.

The first questionnaire was designed to collect demographic and general data about the participants, including age, smoking habits, physical activity, stress exposure, education, occupational activity, and information concerning sexual partners, marriages, deliveries, and miscarriages.

The next questionnaire, FSFI, is a multidimensional, self-reported scale that consists of 19 close-ended questions covering six domains, evaluating all aspects of the female sexual cycle during the past four weeks: sexual desire (questions 1–2), arousal (questions 3–6), lubrication (questions 7–10), orgasm (questions 11–13), satisfaction (questions 14–16) and pain (questions 17–19) [37,38]. Lower scores on any of the subscales indicate worse sexual function. Scoring ranges for items 3–14 and 17–19 are between 0 and 5, while for items 1, 2, 15, and 16 are between 1 and 5 [37]. Domain scores were obtained by summing the individual item scores for each domain, and multiplying the sum by the respective domain factor (0.6 for desire, 0.3 for arousal and lubrication, and 0.4 for orgasm, satisfaction, and pain). A domain score of 0 indicates that the subject had no sexual activity in this period of time. The total FSFI score, which was calculated by summing all six domain scores, may range from 2 to 36 (higher scores indicate better sexual function) [37]. The cut-off FSFI score of 26.55 was used to differentiate women with and without sexual dysfunction [38].

The last questionnaire, the Beck Depression Inventory-Second Edition (BDI-II), is a 21-item self-report inventory designed to measure the presence and severity of depressive symptoms, corresponding with the diagnostic criteria for depressive disorders outlined in the Diagnostic and Statistical Manual of Mental Disorders, Fourth Edition [39,40]. All items are scored on a 4-point Likert scale, ranging from 0 (normal) to 3 (most severe), based on the severity in the last two weeks. The summative score may range from 0 to 63, with a score of 14 or greater typically used to indicate clinically significant depression. The higher the score, the more severe the depressive symptoms [39]. These symptoms were classified as follows: patients with scores below 13 were considered non-depressed, women with scores between 14 and 19 were considered mildly depressed, patients with scores between 20 and 28 were considered moderately depressed, while women with scores between 29 and 63 were considered severely depressed [41].

### 2.5. Quality of Life After 5 Years

Five years after completing study participation, all participants were asked to fill in the abbreviated version of the World Health Organization’s Quality of Life questionnaire (WHOQOL-BREF), which is a 26-item, cross-cultural, and self-administered scale [42,43]. Blank questionnaires were sent by mail together with questions about their actual sociodemographic characteristics and the results of actual biochemical results. Two items (1 and 2) of this questionnaire, labeled as global items, measure the overall perceptions of quality of life and general health. Items 3–26 are used to derive four domain scores: physical health (seven items), psychological health (six items), social relationships (three items), and environment (8 items) [42]. The time span covers the past two weeks. All facets are measured using a five-point Likert scale, and three must be reversed before scoring. Raw domain scores were converted to transformed scores, ranging from 4 to 20. Higher scores indicated a better quality of life [43].

### 2.6. Statistical Analysis

All continuous variables were log transformed to stabilize variance and to handle data points that differ significantly from others. Comparisons between the groups were carried out using either one-way analysis of variance followed by the post hoc Bonferroni test (comparisons between three groups) or two-sample *t* tests for unpaired values (comparisons between two groups). Within-group comparisons were performed using paired Student’s *t*-tests. The chi-square test was used to compare dichotomous variables. The strength and direction of the association between two variables were assessed using Pearson’s r tests (two continuous variables), the phi coefficient (two dichotomous variable), or the point biserial correlation coefficient (one continuous and one dichotomous variable). All two-sided *p*-values below 0.05 were considered to indicate statistical significance.

## 3. Results

### 3.1. The Course of the Study (Table 1)

Of 180 recruited patients, 19 (10.5%) did not complete the study protocol. The reasons for dropping out of the study are summarized in Table 1. Thus, the statistical analysis included 161 patients (89.5%) who completed the study and were classified as adherent to their treatment. The post hoc power calculation showed that the sample size was adequate to obtain a significant result if an effect was present.

**Table 1:** The reasons for dropping out of the study.

Variable	Group I		Group II		Group III	
	Subgroup A	Subgroup B	Subgroup A	Subgroup B	Subgroup A	Subgroup B
**Total number**	2	3	3	5	2	4
**Adverse effects**	1	1	-	-	-	1
**Hospitalization**	-	-	-	1	-	-
**Poor adherence**	1	2	-	-	2	1
**Starting new Chronic treatment**	-	-	1	1	-	2
**Consent withdrawal**	-	-	1	2	-	-
**Lost to follow-up**	-	-	1	1	-	-

Group I: levothyroxine-treated women with PPT and subclinical hypothyroidism; Group II: women with PPT and subclinical hypothyroidism refusing levothyroxine therapy; Group III: levothyroxine-treated women with PPT and overt hypothyroidism. Subgroup A: vitamin D-sufficient women; Subgroup B: vitamin D-deficient/insufficient women.

### 3.2. General Characteristics of the Study Groups (Table 2)

At entry, the study subgroups were similar in terms of age, time since delivery, duration of symptoms, smoking, physical activity, education, occupational activity, type of work, the number of sexual partners, the number and duration of marriages, the number of deliveries and miscarriages, stress exposure, body mass index, and blood pressure. The body mass index and blood pressure did not change during the study.

The daily levothyroxine dose during the study was higher in women with overt than subclinical disease. Vitamin D intake and the percentage of users of vitamin D supplements were higher in vitamin D-sufficient than vitamin D-deficient/insufficient patients.

**Table 2:** General characteristics of the study groups.

Variable	Group I		Group II		Group III	
	Subgroup A	Subgroup B	Subgroup A	Subgroup B	Subgroup A	Subgroup B
**Number of patients**	28	27	27	25	28	26
**Age** (years)	31 ± 6	31 ± 5	29 ± 8	31 ± 8	32 ± 6	31 ± 7
**Time since delivery** (weeks)	32 ± 9	30 ± 7	31 ± 9	29 ± 8	32 ± 10	32 ± 9
**Duration of symptoms** (weeks)	8 ± 5	7 ± 6	7 ± 4	6 ± 4	7 ± 5	7 ± 4
**Smokers** (%)/**Number of cigarettes a day** (n)**/Duration of smoking** (months)	25/11 ± 5/78 ± 28	30/9 ± 6/74 ± 30	26/10 ± 6/72 ± 32	25/9 ± 6/75 ± 26	29/9 ± 5/80 ± 40	27/11 ± 5/77 ± 38
**Physical activity: total/several times a week/once a week/once a month** (%)	61/29/21/11	55/26/18/11	59/26/18/15	60/28/20/12	57/21/21/15	58/23/19/16
**Primary or vocational/secondary/university education** (%)	14/43/43	15/37/48	11/41/48	16/36/48	14/39/47	15/39/46
**Occupational activity**/**Blue-collar/white-collar/pink-collar workers** (%)	68/25/25/18	70/26/30/14	63/22/30/11	64/24/24/16	64/21/25/18	69/23/31/15
**Number of sexual partners** (n)	1.9 ± 0.6	1.7 ± 0.8	1.8 ± 0.7	1.7 ± 0.8	2.0 ± 0.8	1.8 ± 0.7
**Number of marriages** (n)/**duration of marriages** (months)	1.1 ± 0.4/54 ± 19	1.1 ± 0.4/55 ± 19	1.2 ± 0.4/52 ± 20	1.1 ± 0.5/51 ± 19	1.2 ± 0.6/65 ± 118	1.2 ± 0.6/59 ± 20
**Number of deliveries** (n)/**Number of miscarriages** (n)	1.2 ± 0.5/0.7 ± 0.5	1.1 ± 0.5/0.7 ± 0.4	1.2 ± 0.6/0.6 ± 0.4	1.2 ± 0.6/0.8 ± 0.5	1.2 ± 0.5/0.8 ± 0.6	1.3 ± 0.6/0.7 ± 0.5
**Stress exposure** (%)	86	85	81	80	89	85
**Body mass index** (kg/m^2^)	25.1 ± 4.8	25.7 ± 4.2	24.9 ± 4.6	25.2 ± 4.1	26.2 ± 4.3	26.8 ± 5.0
**Systolic blood pressure** (mm Hg)	129 ± 19	127 ± 18	126 ± 18	129 ± 20	128 ± 16	130 ± 15
**Diastolic blood pressure** (mm Hg)	77 ± 6	75 ± 5	76 ± 6	76 ± 5	76 ± 6	77 ± 6
**Daily levothyroxine dose** (µg)	74 ± 14	76 ± 13	-	-	146 ± 26 ^#^	152 ± 25 ^#^
**Total daily vitamin D intake** (µg)	28 ± 9 *	9 ± 3	27 ± 10 *	8 ± 3	27 ± 9 *	8 ± 3
**Users of vitamin D supplements** (%)	43 *	7	37 *	4	39 *	4

Group I: levothyroxine-treated women with PPT and subclinical hypothyroidism; Group II: women with PPT and subclinical hypothyroidism refusing levothyroxine therapy; Group III: levothyroxine-treated women with PPT and overt hypothyroidism. Subgroup A: vitamin D-sufficient women; Subgroup B: vitamin D-deficient/insufficient women. Unlike otherwise stated, the data have been shown as the mean ± standard deviation. * *p* < 0.05 vs. vitamin D-deficient/insufficient women in the same study group, ^#^ *p* < 0.05 vs. women in group I with the same vitamin D status.

### 3.3. Biochemical Variables (Table 3)

Thyroid antibody titers were higher in women with overt than in women with subclinical hypothyroidism, and in the subgroups with vitamin D deficiency/insufficiency than in the subgroups with normal vitamin D status. Levothyroxine reduced TPOAb and TgAb titers in women with thyroid hypofunction and normal vitamin D status. There were no differences between the baseline and follow-up titers of TPOAb and TgAb in levothyroxine-treated women with low vitamin D status and in untreated patients. At the end of the main study, thyroid antibody titers were higher in women with overt than in women with subclinical hypothyroidism, and in untreated than treated women with subclinical hypothyroidism and normal vitamin D status. The percentage changes from baseline differed between levothyroxine-treated and untreated women with normal vitamin D status, and between levothyroxine-treated patients with normal and low vitamin D status.

Baseline 25OHD concentrations were higher in vitamin D-sufficient than vitamin D-deficient/insufficient women, and did not change during the study.

Baseline TSH levels were higher in overt than subclinical hypothyroidism, but there were no within-group differences in this hormone. Levothyroxine treatment reduced TSH levels in both vitamin D-sufficient and vitamin D-deficient/insufficient women. Follow-up TSH levels were higher in untreated than treated women with thyroid hypofunction. There were differences in the percentage changes from baseline in this hormone between levothyroxine-treated women with subclinical and overt hypothyroidism, and between levothyroxine-treated and levothyroxine-naïve women.

At entry, free thyroid hormone levels were higher in women with subclinical than overt thyroid hypofunction. Levothyroxine increased free thyroxine and free triiodothyronine in both vitamin D-sufficient and vitamin D-deficient/insufficient women. Follow-up concentrations were higher in women receiving than not receiving levothyroxine treatment. There were differences in the percentage changes from baseline in both hormones between levothyroxine-treated women with subclinical and overt hypothyroidism, and between levothyroxine-treated and levothyroxine-naïve women.

At baseline, testosterone levels were higher in women with subclinical hypothyroidism than in those with overt disease, as well as higher in vitamin D-sufficient than vitamin D-deficient/insufficient women. Levothyroxine increased testosterone levels only in subgroups with normal vitamin D status. At the end of the main study, testosterone levels were higher in levothyroxine-treated women with normal vitamin D status than in untreated women or levothyroxine-treated women with low vitamin D status. They were also lower in levothyroxine-treated women with overt disease and low vitamin D status than in the remaining subgroups of vitamin D-deficient/insufficient women, and differed between both subgroups of untreated patients. There were differences in the percentage changes from baseline between vitamin D-sufficient individuals with overt and subclinical thyroid hypofunction, between levothyroxine-treated patients with normal vitamin D status and untreated patients, and between levothyroxine-treated patients with different vitamin D status.

Baseline estradiol levels were higher in women with subclinical than overt hypothyroidism, but without within-group differences. Levothyroxine increased this hormone only in both subgroups of women with overt thyroid hypofunction. At the end of the main study, estradiol concentrations in all subgroups were similar. Patients with overt disease differed from the remaining study subgroups in the percentage changes from baseline in estradiol levels.

At entry, prolactin concentrations were higher in women with overt than in women with subclinical hypothyroidism. The decrease in prolactin concentrations was statistically significant only in both subgroups of women with overt hypothyroidism. At the end of the main study, prolactin levels were lower in women receiving levothyroxine than in levothyroxine-naïve ones. There were differences in the percentage changes from baseline between levothyroxine-treated women with subclinical and overt hypothyroidism and untreated individuals, and between levothyroxine-treated women with both forms of thyroid hypofunction.

There were no between-group and within-group differences in baseline and follow-up levels of DHEAS, and in the percentage changes from baseline in this hormone.

**Table 3:** Biochemical characteristics of the study groups.

Variable	Group I		Group II		Group III	
	Subgroup A	Subgroup B	Subgroup A	Subgroup B	Subgroup A	Subgroup B
**TPOAb** (IU/mL)						
*Baseline*	780 ± 230 *	950 ± 250	750 ± 280 *	960 ± 300	1300 ± 620 *^#$^	1900 ± 710 ^#$^
*Follow-up*	570 ± 200 ^$&^	860 ± 280	680 ± 210 *	880 ± 290	930 ± 360 *^#$&^	1700 ± 640 ^#$^
*Percentage change from baseline*	−26 ± 16 *^$^	−11 ± 12	−9 ± 10	−8 ± 8	−31 ± 20 *^$^	−12 ± 15
**TgAb** (IU/mL)						
*Baseline*	650 ± 300 *	970 ± 400	670 ± 320 *	990 ± 340	1300 ± 580 *^#$^	1700 ± 700 ^#$^
*Follow-up*	430 ± 190 ^$&^	900 ± 390	602 ± 290 *	890 ± 380	810 ± 400 *^#$&^	1600 ± 640 ^#$^
*Percentage change from baseline*	−37 ± 20 *^$^	−7 ± 11	−10 ± 15	−10 ± 12	−37 ± 18 *^$^	−6 ± 17
**25OHD** (nmol/L)						
*Baseline*	120 ± 25 *	53 ± 12	120 ± 28 *	51 ± 12	120 ± 23 *	49 ± 13
*Follow-up*	120 ± 27 *	54 ± 10	120 ± 26 *	52 ± 10	120 ± 24 *	52 ± 12
*Percentage change from baseline*	4 ± 10	2 ± 11	0 ± 8	2 ± 8	4 ± 9	6 ± 14
**TSH** (mIU/L)						
*Baseline*	7.6 ± 1.3	7.3 ± 1.2	7.5 ± 1.5	7.4 ± 1.3	19 ± 5.5 ^#$^	20 ± 5.8 ^#$^
*Follow-up*	1.4 ± 0.6 ^$&^	1.6 ± 0.5 ^$&^	7.2 ± 1.8	7.6 ± 2.0	1.3 ± 0.5 ^$&^	1.4 ± 0.5 ^$&^
*Percentage change from baseline*	−82 ± 9 ^$^	−78 ± 11 ^$^	−4 ± 18	3 ± 15	−93 ± 5 ^#$^	−92 ± 5 ^#$^
**Free thyroxine** (pmol/L)						
*Baseline*	14 ± 2.7	14 ± 2.9	14 ± 2.5	14 ± 2.5	7.8 ± 1.1 ^#$^	8.0 ± 1.4 ^#$^
*Follow-up*	18 ± 4.4 ^$&^	19 ± 4.2 ^$&^	14 ± 2.8	14 ± 2.6	19 ± 3.2 ^$&^	18 ± 3.5 ^$&^
*Percentage change from baseline*	32 ± 18 ^$^	35 ± 20 ^$^	−1 ± 10	−2 ± 12	138 ± 58 ^#$^	125 ± 53 ^#$^
**Free triiodothyronine** (pmol/L)						
*Baseline*	3.1 ± 0.61	2.9 ± 0.50	3.0 ± 0.48	3.1 ± 0.62	2.3 ± 0.53 ^#$^	2.3 ± 0.40 ^#$^
*Follow-up*	4.6 ± 1.2 ^$&^	4.5 ± 1.1 ^$&^	2.9 ± 0.61	3.0 ± 0.82	4.4 ± 1.0 ^$&^	4.3 ± 0.89 ^$&^
*Percentage change from baseline*	48 ± 20 ^$^	55 ± 23 ^$^	−3 ± 12	−3 ± 16	91 ± 28 ^#$^	87 ± 25 ^#$^
**Testosterone** (nmol/L)						
*Baseline*	1.4 ± 0.43 *	1.2 ± 0.37	1.4 ± 0.34 *	1.2 ± 0.39	0.98 ± 0.30 *^#$^	0.79 ± 0.34 ^#$^
*Follow-up*	1.7 ± 0.46 *^$&^	1.2 ± 0.32	1.4 ± 0.38 *	1.2 ± 0.42	1.5 ± 0.56 *^$&^	0.81 ± 0.40 ^#$^
*Percentage change from baseline*	18 ± 11 *^$^	−3 ± 15	−3 ± 12	−2 ± 10	55 ± 20 *^#$^	3 ± 20
**Estradiol** (pmol/L)						
*Baseline*	300 ± 100	290 ± 98	300 ± 110	290 ± 110	190 ± 91 ^#$^	170 ± 84 ^#$^
*Follow-up*	360 ± 130	340 ± 140	320 ± 120	310 ± 110	350 ± 130 ^&^	320 ± 120 ^&^
*Percentage change from baseline*	20 ± 40	19 ± 37	5 ± 15	6 ± 20	84 ± 39 ^#$^	89 ± 46 ^#$^
**Prolactin** (mIU/L)						
*Baseline*	600 ± 350	610 ± 360	580 ± 310	600 ± 280	830 ± 390 ^#$^	840 ± 360 ^#$^
*Follow-up*	450 ± 220 ^$^	460 ± 210 ^$^	600 ± 300	610 ± 290	410 ± 190 ^$&^	430 ± 210 ^$&^
*Percentage change from baseline*	−24 ± 30 ^$^	−25 ± 34 ^$^	4 ± 12	2 ± 14	−51 ± 2 ^#$^	−49 ± 23 ^#$^
**DHEAS** (μmol/L)						
*Baseline*	5.0 ± 1.1	4.9 ± 1.0	5.2 ± 1.5	5.0 ± 1.0	4.7 ± 1.4	4.8 ± 1.3
*Follow-up*	5.2 ± 1.0	5.0 ± 1.2	5.1 ± 1.3	4.9 ± 1.1	5.0 ± 1.2	4.8 ± 1.2
*Percentage change from baseline*	4 ± 10	3 ± 12	−3 ± 14	−2 ± 11	6 ± 13	1 ± 8

Group I: levothyroxine-treated women with PPT and subclinical hypothyroidism; Group II: women with PPT and subclinical hypothyroidism refusing levothyroxine therapy; Group III: levothyroxine-treated women with PPT and overt hypothyroidism. Subgroup A: vitamin D-sufficient women; Subgroup B: vitamin D-deficient/insufficient women. The data have been shown as the mean ± standard deviation. Means and standard deviations have been rounded to two significant digits, while percentage values have been rounded to integers. * *p* < 0.05 vs. vitamin D-deficient/insufficient women in the same study group, ^#^ *p* < 0.05 vs. women in group I with the same vitamin D status, ^$^ *p* < 0.05 vs. women in group II with the same vitamin D status, ^&^ *p* < 0.05 vs. baseline value.

### 3.4. Sexual Functioning (Table 4)

At baseline, the overall FSFI score and all domain scores were lower in women with overt hypothyroidism than in women with subclinical hypothyroidism, while the opposite relationship was observed for the percentage of patients with FSD. In each study group, the domain score for desire, but not the remaining scores, was higher in vitamin D-sufficient than vitamin D-deficient/insufficient women. The Levothyroxine treatment of vitamin D-sufficient women with both forms of hypothyroidism increased the total and all domain scores, and decreased the percentage of patients with FSD. In vitamin D-deficient/insufficient women, the drug increased scores for arousal, lubrication, and sexual satisfaction. In patients with low vitamin D status and overt, but not subclinical, disease, the drug increased the total FSFI score, and reduced the percentage of patients with FSD. At the end of the main study, there were no differences between vitamin D-sufficient women with both types of hypothyroidism, and except for desire between both groups of untreated women. Levothyroxine-treated women with normal vitamin D status differed in all assessed parameters from untreated women with normal vitamin D status, and from levothyroxine-treated women with low vitamin D status. There were between-group differences in follow-up values of total and domain scores between the subgroups of vitamin D-deficient/insufficient women. The percentage changes from baseline in all scores were greatest in women with overt disease and normal vitamin D status, more pronounced in women with subclinical disease and normal vitamin D status than in women with subclinical disease and low vitamin D status, and more pronounced in women with subclinical disease and normal vitamin D status than in untreated patients. The impact on the overall FSFI score, arousal, lubrication, and satisfaction was stronger in vitamin D-deficient/insufficient women with overt than with subclinical disease.

**Table 4:** Sexual functioning in the study groups.

Variable	Group I		Group II		Group III	
	Subgroup A	Subgroup B	Subgroup A	Subgroup B	Subgroup A	Subgroup B
**FSFI score**						
*Baseline*	27 ± 3.9	26 ± 3.7	27 ± 4.0	26 ± 3.7	22 ± 3.0 ^#$^	21 ± 2.9 ^#$^
*Follow-up*	32 ± 2.7 *^$&^	28 ± 4.2	27 ± 4.2	25 ± 4.4	32 ± 2.8 *^$&^	24 ± 4.0 ^#&^
*Percentage change from baseline*	20 ± 10 *^$^	7 ± 8 ^$^	−1 ± 7	−2 ± 8	42 ± 12 *^#$^	14 ± 10 ^#$^
**FSFI score ≤ 26.55** (n (%))						
*Baseline*	12 (43)	14 (52)	11 (41)	12 (48)	20 (71) ^#$^	21 (81) ^#$^
*Follow-up*	3 (11) *^$&^	11 (41)	11 (41)	12 (48)	3 (11) *^$&^	15 (58) ^&^
**Sexual desire**						
*Baseline*	4.8 ± 0.65 *	4.3 ± 0.98	4.8 ± 0.98 *	4.2 ± 0.95	3.7 ± 1.0 *^#$^	3.1 ± 1.1 ^#$^
*Follow-up*	5.4 ± 0.48 *^$&^	4.4 ± 0.86	4.7 ± 0.82 *	4.2 ± 1.0	5.4 ± 0.52 *^$&^	3.2 ± 1.2 ^#$^
*Percentage change from baseline*	14 ± 14 *^$^	3 ± 10	−2 ± 10	−1 ± 8	46 ± 18 *^#$^	3 ± 16
**Sexual arousal**						
*Baseline*	4.4 ± 0.82	4.2 ± 0.81	4.5 ± 0.74	4.3 ± 0.79	3.8 ± 1.1 ^#$^	3.5 ± 1.1 ^#$^
*Follow-up*	5.5 ± 0.48 *^$&^	4.8 ± 0.75 ^$&^	4.6 ± 0.88	4.3 ± 0.85	5.5 ± 0.51 *^$&^	4.6 ± 0.76 ^&^
*Percentage change from baseline*	24 ± 14 *^$^	15 ± 11 ^$^	2 ± 11	0 ± 8	44 ± 15 *^#$^	32 ± 14 ^#$^
**Lubrication**						
*Baseline*	4.5 ± 0.85	4.4 ± 0.78	4.6 ± 0.87	4.5 ± 0.89	3.9 ± 1.05 ^#$^	3.7 ± 1.1 ^#$^
*Follow-up*	5.3 ± 0.73 *^$&^	4.9 ± 0.65 ^$&^	4.5 ± 0.92	4.2 ± 0.95	5.4 ± 0.65 *^$&^	4.6 ± 0.83 ^&^
*Percentage change from baseline*	18 ± 10 *^$^	10 ± 12 ^$^	−3 ± 8	−5 ± 11	39 ± 16 *^#$^	22 ± 11 ^#$^
**Orgasm**						
*Baseline*	4.1 ± 0.86	4.0 ± 0.94	4.1 ± 1.00	4.0 ± 0.89	3.4 ± 1.2 ^#$^	3.2 ± 1.1 ^#$^
*Follow-up*	5.4 ± 0.53 *^$&^	3.9 ± 1.1	4.0 ± 0.92	3.9 ± 1.1	5.4 ± 0.60 *^$&^	3.4 ± 1.2 ^#$^
*Percentage change from baseline*	31 ± 18 *^$^	−3 ± 12	−1 ± 14	−2 ± 11	58 ± 20 *^#$^	6 ± 20
**Sexual satisfaction**						
*Baseline*	4.5 ± 0.75	4.5 ± 0.83	4.5 ± 0.80	4.4 ± 0.71	3.9 ± 0.91 ^#$^	3.9 ± 0.95 ^#$^
*Follow-up*	5.4 ± 0.52 *^$&^	4.9 ± 0.74 ^$&^	4.4 ± 0.91	4.2 ± 0.95	5.5 ± 0.50 *^$&^	4.6 ± 0.78 ^&^
*Percentage change from baseline*	20 ± 15 *^$^	10 ± 14 ^$^	−2 ± 13	−4 ± 14	40 ± 20 *^#$^	20 ± 12 ^#$^
**Pain**						
*Baseline*	4.7 ± 0.93	4.5 ± 0.91	4.7 ± 0.88	4.6 ± 0.87	4.1 ± 1.0 ^#$^	3.9 ± 0.94 ^#$^
*Follow-up*	5.3 ± 0.61 *^$&^	4.7 ± 0.72	4.8 ± 0.75	4.6 ± 0.80	5.4 ± 0.47 *^$&^	4.0 ± 0.98 ^#$^
*Percentage change from baseline*	15 ± 12 *^$^	4 ± 10	3 ± 11	2 ± 10	31 ± 14 *^#$^	2 ± 12

Group I: levothyroxine-treated women with PPT and subclinical hypothyroidism; Group II: women with PPT and subclinical hypothyroidism refusing levothyroxine therapy; Group III: levothyroxine-treated women with PPT and overt hypothyroidism. Subgroup A: vitamin D-sufficient women; Subgroup B: vitamin D-deficient/insufficient women. The data have been shown as the mean ± standard deviation. Means and standard deviations have been rounded to two significant digits, while percentage values have been rounded to integers. * *p* < 0.05 vs. vitamin D-deficient/insufficient women in the same study group, ^#^ *p* < 0.05 vs. women in group I with the same vitamin D status, ^$^ *p* < 0.05 vs. women in group II with the same vitamin D status, ^&^ *p* < 0.05 vs. baseline value.

### 3.5. Depressive Symptoms (Table 5)

The BDI-II score, and the percentage of patients with total and mild depressive symptoms were higher in women with overt than in women with subclinical hypothyroidism. Levothyroxine reduced the BDI-II score and the percentage of patients with total and mild depressive symptoms only in subgroups of women with normal vitamin D status. There were no differences between the baseline and follow-up values of these parameters in untreated women. At the end of the study period, the BDI-II score and the percentage of patients with total and mild depressive symptoms were lower in both subgroups of levothyroxine-treated women with normal vitamin D status than in the respective subgroups with low vitamin D status, lower in levothyroxine-treated than untreated women with normal vitamin D status, and higher in vitamin D-deficient/insufficient women with overt than subclinical hypothyroidism. There were differences in the percentage changes from baseline between levothyroxine-treated women with normal and low vitamin D status, and in the percentage changes from baseline between levothyroxine-treated and untreated women with normal vitamin D status.

**Table 5:** Depressive symptoms in the study groups.

Variable	Group I		Group II		Group III	
	Subgroup A	Subgroup B	Subgroup A	Subgroup B	Subgroup A	Subgroup B
**BDI-II score** (mean ± standard deviation)						
*Baseline*	12 ± 2.9	13 ± 2.8	12 ± 3.7	13 ± 3.1	15 ± 3.4 ^#$^	16 ± 3.5 ^#$^
*Follow-up*	10 ± 3.1 *^$&^	13 ± 3.4	13 ± 3.9	13 ± 3.8	11 ± 2.7 *^$&^	16 ± 4.0 ^#$^
*Percentage change from baseline*	−16 ± 10 *^$^	2 ± 6	2 ± 6	2 ± 5	−23 ± 16 *^$^	−1 ± 5
**Depressive symptoms** (n (%))						
*Baseline*	10 (36)	12 (44)	11 (41)	10 (40)	18 (64) ^#$^	18 (69) ^#$^
*Follow-up*	6 (22) *^$&^	12 (44)	12 (44)	11 (44)	8 (29) *^$&^	17 (65) ^#$^
**Mild symptoms** (n (%))						
*Baseline*	10 (36)	12 (44)	11 (41)	9 (36)	17 (60) ^#$^	17 (65) ^#$^
*Follow-up*	6 (22) *^$&^	12 (44)	12 (44)	10 (40)	8 (29) *^$&^	16 (61) ^#$^
**Moderate symptoms** (n (%))						
*Baseline*	0 (0)	0 (0)	0 (0)	1 (4)	1 (4)	1 (4)
*Follow-up*	0 (0)	0 (0)	0 (0)	1 (4)	0 (0)	1 (4)
**Severe symptoms** (n (%))						
*Baseline*	0 (0)	0 (0)	0 (0)	0 (0)	0 (0)	0 (0)
*Follow-up*	0 (0)	0 (0)	0 (0)	0 (0)	0 (0)	0 (0)

Group I: levothyroxine-treated women with PPT and subclinical hypothyroidism; Group II: women with PPT and subclinical hypothyroidism refusing levothyroxine therapy; Group III: levothyroxine-treated women with PPT and overt hypothyroidism. Subgroup A: vitamin D-sufficient women; Subgroup B: vitamin D-deficient/insufficient women. Means and standard deviations have been rounded to two significant digits, while percentage values have been rounded to integers. * *p* < 0.05 vs. vitamin D-deficient/insufficient women in the same study group, ^#^ *p* < 0.05 vs. women in group I with the same vitamin D status, ^$^ *p* < 0.05 vs. women in group II with the same vitamin D status, ^&^ *p* < 0.05 vs. baseline value.

### 3.6. Quality of Life Five Years After the Main Study (Table 6)

Ninety-seven responders (60.82% of all patients participating in the main study) sent back completed WHOQOL-BREF questionnaires together with answers to our questions. At the time of this questionnaire analysis, there were no between-subgroup differences in the percentage of patients treated with levothyroxine and in the percentage of patients with low vitamin D status.

Scores for overall perception of quality of life and overall perception of general health, and domain scores for physical and psychological health, but not for the remaining two domains, were higher in responders treated during the main study with levothyroxine because of overt or subclinical thyroid hypofunction and having normal vitamin D status than in levothyroxine-treated women with low vitamin D status and in women who had not been receiving thyroid hormone substitution.

**Table 6:** Quality of life five years after the study.

Variable	Group I		Group II		Group III	
	Subgroup A	Subgroup B	Subgroup A	Subgroup B	Subgroup A	Subgroup B
**Number of responders**	17	17	16	15	17	15
**Levothyroxine treatment** (%)	24	29	31	27	29	27
**Low vitamin D status** (%)	47	53	50	53	47	53
**Overall perception of quality of life**	4.5 ± 0.46 *^$^	3.9 ± 0.58	3.6 ± 0.74	3.8 ± 0.69	4.4 ± 0.51 *^$^	3.9 ± 0.71
**Overall perception of general health**	4.5 ± 0.42 *^$^	4.0 ± 0.64	4.0 ± 0.68	3.8 ± 0.62	4.5 ± 0.48 *^$^	4.0 ± 0.55
**Physical health**	16 ± 2.3 *^$^	14 ± 2.6	14 ± 3.5	13 ± 4.1	16 ± 2.5 *^$^	13 ± 4.0
**Psychological health**	15 ± 4.2 *^$^	12 ± 3.2	12 ± 4.0	12 ± 3.8	16 ± 3.9 *^$^	13 ± 3.2
**Social relationship**	16 ± 2.9	15 ± 3.2	15 ± 3.5	14 ± 3.9	15 ± 4.0	14 ± 3.7
**Environment**	15 ± 3.2	14 ± 3.6	14 ± 4.1	14 ± 4.0	15 ± 3.4	15 ± 3.9

Group I: levothyroxine-treated women with PPT and subclinical hypothyroidism during the main study; Group II: women with PPT and subclinical hypothyroidism refusing levothyroxine therapy during the main study; Group III: levothyroxine-treated women with PPT and overt hypothyroidism during the main study. Subgroup A: vitamin D-sufficient women during the main study; Subgroup B: vitamin D-deficient/insufficient women during the main study. Overall perception of quality of life and overall perception of general health are scored from 1 to 5, while physical health, psychological heath, social relationship, and environment are scored from 4 to 20 (higher scores indicate a better quality of life). Unlike otherwise stated, the data have been shown as the mean ± standard deviation. Means and standard deviations have been rounded to two significant digits. * *p* < 0.05 vs. vitamin D-deficient/insufficient women in the same study group, ^$^ *p* < 0.05 vs. women in group II with the same vitamin D status.

### 3.7. Correlations (Table 7, Table 8 and Table 9)

The impact of levothyroxine on all aspects of sexual functioning correlated with the effect of this agent on TPOAb titers, on free thyroxine levels, and in case of libido and arousal with the impact on testosterone (Table 7). The changes in sexual functioning did not correlate with the changes in TgAb, TSH, free thyroxine, estradiol, prolactin, and DHEAS. Moreover, there were correlations between the impact of levothyroxine on TPOAb titers and free triiodothyronine levels and their baseline values, and in vitamin D-sufficient women between the impact of levothyroxine on testosterone and on TPOAb (Table 7).

**Table 7:** Correlations between sexual functioning and biochemical variables in levothyroxine-treated women with PPT and hypothyroidism.

Correlated Variables		Group I		Group III	
		Subgroup A	Subgroup B	Subgroup A	Subgroup B
Δ Overall FSFI score	Δ TPOAb	0.42 ^b^	0.35 ^a^	0.36 ^b^	0.31 ^a^
Δ Sexual desire	Δ TPOAb	0.38 ^b^	0.32 ^a^	0.36 ^b^	0.32 ^a^
Δ Sexual arousal	Δ TPOAb	0.35 ^a^	0.32 ^a^	0.38 ^b^	0.31 ^a^
Δ Lubrication	Δ TPOAb	0.41 ^b^	0.30 ^a^	0.35 ^a^	0.26 ^a^
Δ Orgasm	Δ TPOAb	0.42 ^b^	0.34 ^a^	0.39 ^b^	0.39 ^b^
Δ Sexual satisfaction	Δ TPOAb	0.46 ^c^	0.36 ^b^	0.44 ^b^	0.39 ^b^
Δ Pain	Δ TPOAb	0.44 ^b^	0.29 ^a^	0.42 ^b^	0.31 ^a^
Δ Overall FSFI score	Δ Free triiodothyronine	0.41 ^b^	0.45 ^b^	0.41 ^b^	0.34 ^a^
Δ Sexual desire	Δ Free triiodothyronine	0.46 ^c^	0.43 ^b^	0.39 ^b^	0.31 ^a^
Δ Sexual arousal	Δ Free triiodothyronine	0.47 ^c^	0.39 ^b^	0.42 ^b^	0.43 ^b^
Δ Lubrication	Δ Free triiodothyronine	0.43 ^b^	0.41 ^b^	0.47 ^c^	0.42 ^b^
Δ Orgasm	Δ Free triiodothyronine	0.41 ^b^	0.50 ^c^	0.47 ^c^	0.31 ^a^
Δ Sexual satisfaction	Δ Free triiodothyronine	0.41 ^b^	0.35 ^a^	0.42 ^b^	0.48 ^c^
Δ Pain	Δ Free triiodothyronine	0.40 ^b^	0.38 ^b^	0.42 ^b^	0.43 ^a^
Δ Sexual desire	Δ Testosterone	0.42 ^b^	0.18	0.48 ^a^	0.18
Δ Sexual arousal	Δ Testosterone	0.40 ^b^	0.16	0.42 ^b^	0.16
Δ TPOAb	TPOAb	0.56 ^c^	0.39 ^b^	0.57 ^c^	0.40 ^b^
Δ Free triiodothyronine	Free triiodothyronine	0.39 ^b^	0.31 ^a^	0.39 ^b^	0.32 ^a^
Δ Testosterone	Δ TPOAb	0.44 ^b^	0.39 ^a^	0.43 ^b^	0. 37 ^a^

Group I: levothyroxine-treated women with PPT and subclinical hypothyroidism; Group III: levothyroxine-treated women with PPT and overt hypothyroidism. Subgroup A: vitamin D-sufficient women; Subgroup B: vitamin D-deficient/insufficient women. The data represent the correlation coefficients (r values). ^a^ *p* < 0.05, ^b^ *p* < 0.01, ^c^ *p* < 0.001.

In levothyroxine-treated women with vitamin D-deficiency/insufficiency, but not in women with normal vitamin D status, the impact on the overall FSFI score and on all domain scores inversely correlated with 25OHD levels (Table 8). There were no correlations between sexual functioning of levothyroxine-treated patients and vitamin D intake, the percentage of users of vitamin D supplements and duration of vitamin D supplementation.

**Table 8:** Correlations between the impact of levothyroxine on sexual functioning and 25OHD concentrations in vitamin D deficient/insufficient women with PPT and hypothyroidism.

Correlated Variables		Subgroup IB (Subclinical Hypothyroidism and Low Vitamin D Status)	Subgroup IIIB (Overt Hypothyroidism and Low Vitamin D Status)
Δ FSFI score	25OHD	−0.41 ^b^	−0.43 ^b^
Δ Sexual desire	25OHD	−0.52 ^c^	−0.55 ^c^
Δ Sexual arousal	25OHD	−0.40 ^b^	−0.36 ^b^
Δ Lubrication	25OHD	−0.31 ^a^	−0.40 ^b^
Δ Orgasm	25OHD	−0.42 ^b^	−0.44 ^b^
Δ Sexual satisfaction	25OHD	−0.32 ^a^	−0.32 ^a^
Δ Pain	25OHD	−0.40 ^b^	−0.42 ^b^

The data represent the correlation coefficients (r values). ^a^ *p* < 0.05, ^b^ *p* < 0.01, ^c^ *p* < 0.001.

In levothyroxine-treated patients (groups I and III), the changes in the BDI-II score correlated with the changes in all aspects of sexual functioning. These correlations were strongest for desire and arousal (Table 9). The changes in the BDI-II score did not correlate with hormone levels, antibody titers, and vitamin D status.

**Table 9:** Correlations between the impact of levothyroxine on sexual functioning and on depressive symptoms in women with PPT and hypothyroidism.

Correlated Variables		Group I		Group III	
		Subgroup A	Subgroup B	Subgroup A	Subgroup B
Δ BDI-II	Δ FSFI score	0.40 ^b^	0.32 ^a^	0.40 ^b^	0.34 ^a^
Δ BDI-II	Δ Sexual desire	0.59 ^c^	0.42 ^b^	0.58 ^c^	0.43 ^b^
Δ BDI-II	Δ Sexual arousal	0.50 ^c^	0.42 ^b^	0.51 ^c^	0.41 ^b^
Δ BDI-II	Δ Lubrication	0.38 ^b^	0.36 ^b^	0.39 ^b^	0.37 ^b^
Δ BDI-II	Δ Orgasm	0.37 ^b^	0.32 ^a^	0.35 ^a^	0.29 ^a^
Δ BDI-II	Δ Sexual satisfaction	0.36 ^b^	0.36 ^b^	0.36 ^b^	0.35 ^b^
Δ BDI-II	Δ Pain	0.40 ^b^	0.30 ^a^	0.32 ^a^	0.29 ^a^

Group I: levothyroxine-treated women with PPT and subclinical hypothyroidism; Group III: levothyroxine-treated women with PPT and overt hypothyroidism. Subgroup A: vitamin D-sufficient women; Subgroup B: vitamin D-deficient/insufficient women. The data represent the correlation coefficients (r values). ^a^ *p* < 0.05, ^b^ *p* < 0.01, ^c^ *p* < 0.001.

Follow-up values of the total FSFI score (r values between 0.295 [*p* = 0.048] and 0.43 [*p* < 0.001]), but not antibody titers, hormone levels and the BDI-II score during the main study, correlated with the overall perception of quality of life, overall perception of general health, physical health and psychological health five years later.

## 4. Discussion

The obtained results allow us to draw some novel conclusions. Firstly, the degree of hypothyroidism determines sexual functioning of women with PPT. The total FSFI score and all domain scores were lower in individuals with overt than with subclinical hypothyroidism. Interestingly, within each treatment group, vitamin D-deficient/insufficient women were characterized by lower scores for desire than vitamin D-sufficient ones. This finding is in line with our previous observations showing that isolated vitamin D deficiency and isolated vitamin D insufficiency were accompanied by impaired sexual drive, while the former also by deterioration of orgasm and sexual satisfaction [9]. The study groups were intentionally matched for age, body mass index, and blood pressure because thyroid hypofunction may be complicated by an increase in body weight and hypertension [44], and because all these factors were found to affect female sexual function [45,46,47]. Similarly, the obtained results cannot be attributed to other differences in the baseline patients’ characteristics, which was similar in all study subgroups, and, because of the exclusion criteria, they cannot be regarded as a consequence of concomitant disorders or administration of other agents. Thus, it seems that thyroid hypofunction and low vitamin D status have additive effects on women’s sexual health in PPT, and that their coexistence may make patients with PPT particularly prone to sexual dysfunction.

Another novel finding of our study is that levothyroxine improved sexual functioning in women with both types of hypothyroidism, and that the strength of this effect was determined by the degree of reduction in TPOAb and an increase in free triiodothyronine in response to chronic levothyroxine administration. Most individuals with thyroid hypofunction on levothyroxine replacement therapy have higher circulating levels of free thyroxine than healthy euthyroid subjects, and to normalize TSH concentration, thyroxine levels often have to reach the upper limit of normal [48]. TSH secretion depends mainly on pituitary conversion of thyroxine to triiodothyronine by type 2 deiodinase, and therefore sensitivity of thyrotrophs to thyroid hormones differs from that of extra-pituitary tissues [49]. These findings well explain why the improvement in female sexual response depended only on the changes in free triiodothyronine, and not on the changes in TSH and free thyroxine. Because the impact on TPOAb and free triiodothyronine correlated with their baseline values, the most impressive improvement in female sexual response seems to occur in women with the most severe forms of PPT, who are characterized by the most abundant inflammatory cell infiltration and the lowest thyroid hormone production. Consequently, this group of patients may benefit particularly from levothyroxine substitution. Our findings cannot be explained by the spontaneous resolution of PPT or by physiological changes in sexual behavior in the first postpartum year. In women refusing vitamin D therapy, the decrease in thyroid antibody titers and in TSH during the six-month follow-up period was not observed or did not reach statistical significance, and, even more importantly, these changes were not accompanied by better sexual functioning. Moreover, the mean time from delivery to the onset of the study was 7–8 months, while the sexual functioning of non-lactating healthy women did not change since the third postpartum month [50]. Thus, our findings are an argument for thyroid hormone substitution in all hypothyroid women with PPT and impaired sexual functioning, even if this disorder is characterized by asymptomatic or oligosymptomatic course.

The most important finding of the present study was that vitamin D status determined levothyroxine action on female sexual functioning in both types of hypothyroidism. In women with normal vitamin D status, the drug improved global sexual functioning and all its aspects, while in the case of low vitamin D status, levothyroxine action was limited to a small increase in arousal, lubrication, and sexual satisfaction. The obtained results suggest that weaker effects in vitamin D deficient/insufficient women can be explained by chronically low availability of vitamin D in this group of women. In line with our explanation, the impact of levothyroxine on both global sexual functioning and all domain scores of the FSFI questionnaire in women with vitamin D deficiency/insufficiency inversely correlated with 25OHD concentration. The lack of similar correlations in women with normal vitamin D status may indicate that serum 25OHD levels in the range between 75 and 200 nmol/L are required to normalize the sexual response of hypothyroid women with PPT to levothyroxine replacement therapy. Moreover, the obtained results indirectly suggest that the currently recommended threshold concentrations for 25OHD (75 nmol/L) are fully justified [29], but they do not allow to recommend an optimal target for 25OHD levels which should be the aim of supplementation. Another important finding is that between-group differences in female sexual functioning do not seem to be associated with the impact on liberation, absorption, distribution, metabolism, and excretion of levothyroxine because there were no correlations between the strength of levothyroxine action and vitamin D intake with supplements and/or the duration of this supplementation. Moreover, an interval of approximately 12 h between taking both drugs minimized the risk of pharmacokinetic interactions between the ingredients of the levothyroxine tablets and of the vitamin D capsules.

It seems that between-group differences in desire and arousal are partially mediated by changes in testosterone production. Baseline levels of this hormone were lower in women with overt than in women with subclinical hypothyroidism, and, independently of the severity of thyroid failure, they were lower in patients with low rather than in those with normal vitamin D status. Levothyroxine treatment increased testosterone levels in women with subclinical and overt hypothyroidism and normal, but not low, vitamin D status, which was paralleled by the improvement in libido only in individuals with 25OHD levels in the range between 75 and 200 nmol/L. Lastly, desire and arousal are controlled by testosterone to a greater extent than the remaining phases of the female sexual response cycle [51,52], which was supported by positive correlations between the improvement in libido and excitement and the impact of levothyroxine on testosterone levels. It is worth mentioning that the increase in testosterone might have been secondary to the improvement in thyroid autoimmunity. In line with this finding, the increase in testosterone levels positively correlated with a levothyroxine-induced decrease in TPOAb titers but not with the changes in TSH and free thyroid hormones. Another argument supporting this explanation are the differences in testosterone levels between various groups of women with autoimmune thyroiditis in whom TSH, free thyroxine, and free triiodothyronine were within the reference range, but differed in thyroid antibody titers [11]. It seems that vitamin D and thyroid autoimmunity may exert the opposite effects at the level of testosterone production. Korean authors reported that vitamin D may increase fertility in healthy non-obese women through the modulation of androgen activity. Similarly to our study, they observed the presence of positive correlations between 25OHD and testosterone and the free androgen index [53]. Considering the presence of the vitamin D receptor in ovarian theca cells [54] and their role in the production of aromatizable androgens [53], it is probable that vitamin D and/or its active metabolites may increase ovarian testosterone production, as it was documented in human testicular cell cultures [55,56].

Unlike testosterone, our study does not provide evidence that the remaining hormones play a role in the impact of levothyroxine on sexual functioning in women with PPT and on the interaction between levothyroxine and vitamin D status. The drug reduced prolactin levels and increased estradiol concentrations in women with overt disease, but both effects probably reflected causal relationships between more severe forms of hypothyroidism and prolactin excess and estradiol deficiency. This explanation is based on the fact that both hyperprolactinemia [36] and decreased estrogen production [57] are complications of untreated or undertreated thyroid hypofunction. However, the improvement in sexual functioning was observed even in women with subclinical hypothyroidism, in whom the impact of treatment on both hormones was neutral. Moreover, even in women with overt hypothyroidism, there were no correlations between the strength of levothyroxine action and levothyroxine-induced changes in prolactin and estradiol. Similarly, despite theoretical premises [58], our findings cannot be explained by the impact on adrenal androgen production. In all study groups, levothyroxine did not affect DHEAS levels, and the effect of treatment on this marker in individual patients did not correlate with the parameters assessed in the FSFI questionnaire.

Based on the obtained results, some clinically relevant conclusions can be drawn. Firstly, even the complete normalization of the hypothalamic–pituitary–thyroid axis activity in hypothyroid patients with PPT may not be sufficient for the normalization of female sexual response in the case of low vitamin D status. Secondly, sexual dysfunction persisting despite effective levothyroxine treatment seems to justify evaluation of vitamin D status. Thirdly, it may be reasonable to measure 25OHD in all sexually active women with PPT who are candidates to receive thyroid hormone supplementation. Fourthly, vitamin D-deficient/insufficient women with PPT should receive levothyroxine in combination with exogenous vitamin D. Fifthly, considering the positive correlations between the impact on sexual functioning and the degree of reduction in TPOAb, it is possible that women with PPT and the highest thyroid antibody titers and normal vitamin D status may benefit even more if levothyroxine is administered in combination with selenomethionine or myo-inositol, non-hormonal agents found to improve female sexual response [11]. Lastly, both overall and domain scores of the FSFI questionnaires depended on TPOAb, but not TgAb, suggesting that only TPOAb titers are able to predict the improvement in women’s sexual health. Thus, a simultaneous assessment of TgAb titers does not have any additive value, and seems unnecessary from a sexual point of view.

The effect of levothyroxine on sexual functioning in vitamin D-sufficient women was accompanied by an improvement in depressive symptoms, which was absent in their vitamin D-deficient/insufficient peers. This finding suggests that the impact of levothyroxine on mood in PPT is determined by the vitamin D status of women, but, considering the lack of correlations between depressive symptoms and 25OHD, this modulatory effect is probably indirect. Although our study is the first one assessing the impact of this agent on the BDI-II score in PPT, differences in vitamin D status may partially explain why the improvement in mood was observed in many, but not in all, hypothyroid individuals chronically treated with levothyroxine [59,60]. It should be emphasized that global sexual functioning and all its aspects were the only variables assessed in the current study that correlated with the overall BDI-II score. Thus, we safely assume that differences in the impact of levothyroxine on depressive symptoms in women with PPT and different vitamin D status were at least in part secondary to differences in sexual functioning. What is more, comparing the strength of correlations, it seems that the levothyroxine-induced improvement in mood in vitamin D-sufficient women reflected to a greater degree the stimulatory effect on libido and excitement than the effect on the remaining dimensions of women’s sexual health. No changes in the body mass index, matching the subgroups for the body mass index and the lack of correlation between the effect on this parameter and on the BDI-II score, contrasting with the presence of such correlations in women with thyroidectomy-induced hypothyroidism [6], indicate that our findings do not seem to be associated with the impact on self-perception of weight. It is, however, worth mentioning that the study design does not allow to exclude that the cause–effect relationship is bidirectional, and that mood improvement may contribute to better sexual functioning. The possibility of reverse causality is backed up by findings suggesting that the improvement in mood is a direct consequence of levothyroxine action and its interaction with vitamin D status. It has been well documented that depressive symptoms associated with thyroid hypofunction often resolve after the restoration of euthyroidism with thyroid hormone replacement therapy [59,61]. Moreover, levothyroxine used as an adjunctive therapy to antidepressants was superior to antidepressants alone in improving mood, and was reported to bring benefits to some patients with treatment-resistant depression, even if they had normal thyroid function [59,62]. There is also ample literature on the association between vitamin D status and depression. Vitamin D receptors and 1-*α*-hydroxylase expression have been detected in brain regions playing a role in the pathophysiology of depression (the hypothalamus, hippocampus, and prefrontal cortex) [63]. Circulating 25OHD levels were significantly inversely associated with the occurrence and severity of depression [64]. Lastly, randomized controlled studies and several meta-analyses showed that vitamin D was efficient in alleviating the symptoms of depression [65,66]. Although the mechanistic explanation of our findings remains unclear, the association between the changes in sexual functioning and depressive symptoms may be mediated by chronic systemic inflammation and/by serotoninergic transmission. An increased production of proinflammatory cytokines and impaired serotoninergic transmission are postulated to have a negative effect on sexual functioning [67,68] and to lead to mood disorders [69,70]. Both these biochemical abnormalities were reported in postpartum women with autoimmune disorders [71,72]. In turn, the opposite effect on central serotoninergic transmission and the proinflammatory state was caused by levothyroxine [73,74].

To assess whether the impact on sexual functioning and depressive symptoms have long-term consequences, we investigated the patients’ quality of life 5 years after the final study visit. Interestingly, the overall perception of quality of life and general health, and the physical and psychological dimensions of quality of life were better in levothyroxine-treated women who underwent PPT than in levothyroxine-naïve ones, as well as in individuals with normal rather than low vitamin D status. Moreover, the global scores correlated with the total FSFI score at the end of the main study, but not with the remaining outcome measures. Undoubtedly, the value of the obtained results is limited by the fact that only about 60% of patients sent back the completed questionnaires, making the analysis underpowered to detect some meaningful differences. Moreover, 5 years later, the population was less homogenous than during the main study (only 24–29% of women continued levothyroxine treatment, the treatment was present in 27–31% initially untreated patients, approximately 20% of responders reported following pregnancies, biochemical parameters were measured using different methods, and only some patients had recent results). However, this analysis provides new pieces of information on PPT and its treatment. Firstly, sexual dysfunction in women with untreated PPT may have an unfavorable impact on later quality of life. Secondly, the degree of wellbeing does not seem to be directly influenced by thyroid autoimmunity, hypothalamic–pituitary–thyroid axis activity, or resultant changes in other hormones and depressive symptoms. Thirdly, levothyroxine treatment of this disease may prevent worsening quality of life, and this effect is particularly noticeable for physical and psychological domains. Lastly, the beneficial effect of thyroid hormone replacement therapy on later quality of life requires normal vitamin D homeostasis.

Several study limitations warrant caution in interpreting the findings. The relatively small sample size and the lack of randomization limit the generalizability of the results, increase the probability of committing a type II error, and increase the risk of selection bias. The responses to the questionnaire questions might have been impacted by subjectivity, human error, or intentional misrepresentation. The study design does not allow us to draw conclusions about sexual functioning and depressive symptoms in breastfeeding women on levothyroxine replacement therapy, because ongoing or recently completed lactation was one of the exclusion criteria. It is difficult to conclude whether the impact on levothyroxine action differs between vitamin D deficiency and vitamin D insufficiency because women with low vitamin D status belonged to the same subgroups. Obligatory iodine prophylaxis in Poland and suboptimal selenium status in women inhabiting the Upper Silesia [75,76] cause that the relationship between levothyroxine action and vitamin D status does not have to be similar in case of limited iodine intake and/or adequate or excess selenium intake. We cannot completely exclude the possibility that sexual functioning and mood might have improved spontaneously. Lastly, although we tried to take precautions at the design stage, our results might have been impacted by regression to the mean (averaging out extreme values in subsequent measurements) and/or regression dilution (resulting from long-term physiological changes in the outcome variables) [77,78].

## 5. Conclusions

The levothyroxine treatment of women with PPT is associated with the improvement in female sexual functioning. The beneficial impact of thyroid hormone supplementation, although observed independently of the severity of thyroid hypofunction, is more pronounced in overt than in subclinical disease. The strength of levothyroxine action on women’s sexual health is determined by vitamin D availability, and is less pronounced in the case of inadequate vitamin D status. In addition to immunosuppressive properties and the impact on thyroid function, the improvement in sexual functioning is partially associated with the levothyroxine-induced increase in testosterone levels. Differences in the impact on serum testosterone may also partially explain various effects of levothyroxine in patients with normal and impaired vitamin D homeostasis. Differences in sexual functioning between women with various vitamin D status seem to be paralleled by similar differences in mood. The obtained results suggest for the first time that low vitamin D status may attenuate levothyroxine action on female sexual function and depressive symptoms in women with hypothyroidism caused by PPT. Because of the lack of similar data and study design limitations, our findings require confirmation in larger-scale controlled trials.

## Data Availability

The data that support the findings of this study are available from the corresponding author upon reasonable request. The data are not publicly available due to privacy and legal restrictions.

## Abbreviations

25OHD	25-hydroxyvitamin D
BDI-II	Beck Depression Inventory-Second Edition
DHEAS	dehydroepiandrosterone sulfate
FSD	Female sexual dysfunction
FSFI	Female Sexual Function Index
PPT	postpartum thyroiditis
TgAb	thyroglobulin antibodies
TPOAb	thyroid peroxidase antibodies
TSH	thyroid-stimulating hormone
WHOQOL-BREF	the World Health Organization’s Quality of Life questionnaire—abbreviated version
